# Optimizing acute ischemic stroke outcome prediction by integrating radiomics features of DSC-PWI and perfusion parameter maps

**DOI:** 10.3389/fneur.2025.1528812

**Published:** 2025-03-21

**Authors:** Huihui Yang, Yingwei Guo, Jiaxi Lu, Haseeb Hassan, Anbo Cao, Yingjian Yang, Mazen M. Yassin, Asim Zaman, Xueqiang Zeng, Xiaoqiang Miao, Ziran Chen, Guangtao Huang, Taiyu Han, Huiling Qiu, Yu Luo, Yan Kang

**Affiliations:** ^1^Country School of Applied Technology, Shenzhen University, Shenzhen, China; ^2^College of Health Science and Environmental Engineering, Shenzhen Technology University, Shenzhen, China; ^3^School of Electrical and Information Engineering, Northeast Petroleum University, Daqing, China; ^4^Department of Radiological Research and Development, Shenzhen Lanmage Medical Technology Co., Ltd, Shenzhen, China; ^5^School of Biomedical Engineering, Shenzhen University Medical School, Shenzhen University, Shenzhen, China; ^6^College of Medicine and Biological Information Engineering, Northeastern University, Shenyang, China; ^7^College of Pharmacy, Shenzhen Technology University, Shenzhen, China; ^8^Department of Radiology, Shanghai Fourth People’s Hospital Affiliated to Tongji University School of Medicine, Shanghai, China; ^9^Engineering Research Centre of Medical Imaging and Intelligent Analysis, Ministry of Education, Shenyang, China

**Keywords:** acute ischemic stroke, DSC-PWI sequence, perfusion parameters, radiomics, prognosis prediction

## Abstract

**Introduction:**

Accurate prediction of the prognostic outcomes for patients with ischemic stroke can contribute to personalized treatment decisions and improve life-saving outcomes. This study focuses on the performance of critical moments DSC-PWI in the prognostic prediction of acute ischemic stroke (AIS). It aims to integrate this with perfusion parameters to enhance prediction accuracy.

**Methods:**

Firstly, The radiomics technique employed to extract DSC-PWI features of critical moments and perfusion parameter features. Following this, a T-test and Lasso algorithm was used to select features associated with the prognosis. Subsequently, machine learning techniques were applied to predict the predictive outcomes of AIS patients.

**Results:**

The experimental results showed that DSC-PWI sequences at three critical time points—the first moment after contrast injection, the moment of minimum mean time intensity, and the last moment, collectively referred to as 3PWI, had better prognostic prediction than a single perfusion parameter, achieving an optimal model AUC of 0.863. The performance improved by 23.9, 19.6, 6, and 24% compared with CBV, CBF, MTT, and Tmax parameters. The best prognostic prediction for AIS was obtained by integrating the radiomic features from both 3PWI and perfusion parameters, resulting in the highest AUC of 0.915.

**Discussion:**

Integrating the radiomics features of DSC-PWI sequences of three critical scan time points with those from perfusion parameters can further improve the accuracy of prognostic prediction for AIS patients. This approach may provide new insights into the prognostic evaluation of AIS and provide clinicians with valuable support in making treatment decisions.

## 1 Introduction

Acute Ischemic Stroke (AIS) is a sudden blockage in the intracranial blood vessels that disrupts the brain’s blood circulation. This disruption leads to varying degrees of tissue damage and necrosis in the affected area, forming two key regions: the infarct core and the ischemic penumbra (1). The infarct core causes brain tissue necrosis due to prolonged ischemia and hypoxia, resulting in irreversible damage (2). In the ischemic penumbra, brain cells retain partial function due to limited blood flow supplied by collateral vessels. However, if the blood supply continues to be insufficient, the cells may gradually die. Therefore, timely reperfusion of the blood vessels in the ischemic penumbra is crucial and is expected to improve patient outcomes. However, there may be a certain degree of prognostic risk such as hemorrhage (3). Therefore, individualized treatment strategies are necessary for patient recovery. Accurately predicting a patient’s prognosis can help physicians more accurately evaluate the treatment effect, make individualized treatment decisions for patients, and reduce the risk of poor treatment outcomes.

In clinical analysis, the modified Rankin scale (mRS) is clinicians’ most commonly used metric to report overall disability in stroke patients. It has been formally recommended for use in acute stroke clinical trials by regulatory agencies and clinical trial methodology consensus groups (4). The scale was adapted by Charles Warlow and others from the Rankin Scale in the 1980s (5). It measures a patient’s ability to live independently, encompassing physical function, mobility, and participation in daily life. A score of 0–2 is often considered a good prognosis; a score of 3–6 is a poor prognosis (6, 7). Although there are clear definitions for each level, the specific scores are derived from the physician’s experience by asking the patient through telephone follow-up, which is highly subjective, and the telephone questioning needs to rely on the patient’s self-representation, which may be subject to bias. These potential factors can affect physicians’ assessment of the prognosis of AIS patients. Thus, a more objective means of analyzing the prognosis of AIS is needed.

In terms of examination cost and scanning time, computed tomography perfusion (CTP) has long been considered the most suitable choice for patients with acute stroke. However, with advancements in MRI speed, concerns about radiation exposure and the use of iodinated contrast agents (which are contraindicated in patients with a history of allergic reactions or renal insufficiency) have prompted considerations of alternative imaging methods (8). Dynamic Susceptibility Contrast-Perfusion Weighted Imaging (DSC-PWI) is currently one of the most commonly used PWI techniques for assessing cerebral perfusion (9). Its fundamental principle relies on the local magnetic field inhomogeneity induced by a paramagnetic contrast agent as it passes through cerebral vasculature after intravenous injection. This leads to varying degrees of T2* signal attenuation proportional to the concentration of the contrast agent, allowing dynamic monitoring of signal intensity to reflect cerebral blood flow changes. DSC-PWI offers a temporal resolution of approximately 0.5–2 s, which is significantly higher than that of Arterial Spin Labeling (ASL), typically ranging from 1 to 4 s. This higher temporal resolution enables real-time, detailed recording of the T2* signal decay process and facilitates the capture of rapid cerebral blood flow changes. By applying mathematical modeling to signal variations allows obtaining the perfusion parameter maps, including Cerebral Blood Volume (CBV), Cerebral Blood Flow (CBF), Mean Transit Time (MTT), Time to Peak (TTP), and Time to Peak of Residual Function (Tmax). These parameters provide information about cerebral blood supply and perfusion, which is critical for understanding brain function, disease diagnosis, and therapeutic regimens development. Many current studies have explored the correlation between perfusion parameter maps and prognostic outcomes in AIS. For example, Park et al. (10) demonstrated that a reduction in rCBV ratio was associated with a poor prognosis in AIS in 58 patients undergoing intravenous thrombolysis, and Schaefer et al. (11) found that an MTT lesion of less than 50 mL had a better performance in predicting a good prognosis for patients. The results of these studies confirm the potential of perfusion parameters in the prognostic prediction of AIS. However, they have analyzed prognosis through statistical methods, with few studies utilizing radiomics features of perfusion parameters to construct prognostic models.

In addition to perfusion parameter maps, numerous studies have utilized information from CT and MRI images to construct prognostic prediction models for AIS through machine learning (ML) and deep learning (DL) methods (12–14). However, few studies have analyzed the ability of 4D perfusion sequences for AIS prognostic prediction. Meng et al. (15) generated four perfusion parameters (CBV, CBF, MTT, and TTP) from PWI to predict the prognosis of AIS patients with or without hemorrhage. After combining the clinical factors, the prediction accuracy reached 89.4%. The generated perfusion parameter maps are more targeted to utilize the cerebral hemodynamic information but ignore the time-dimensional and rich anatomical information of the 4D perfusion sequence. Some researchers have also used spatiotemporal convolutional networks to obtain the time-dimensional features of the 4D CTP (16). However, the features obtained with DL techniques cannot provide the corresponding meanings and will suffer from the problem of poor interpretability. Guo et al. (17) extracted whole-brain Radiomics features for all sequences of DSC-PWI to prognostic prediction of AIS, and the best Area Under the Curve (AUC) obtained was 82.8%. Although this way of analyzing all sequence features ensured comprehensive information, it also brought about problems of high computational volume and tedious tasks. In summary, to ensure the preservation of some temporal information while reducing the computational cost, the present study took radiomics technology to feature quantification of the DSC-PWI sequences at three representative time points as well as the perfusion parameters obtained by post-processing and then constructed a prognostic prediction model for AIS using the ML model, which is expected to become a new clinical auxiliary tool.

In conclusion, this study investigated the roles of DSC-PWI sequences at different scanning time points. It also assessed the impact of individual perfusion parameters (Cerebral Blood Volume [CBV], Cerebral Blood Flow [CBF], Mean Transit Time [MTT], and Time to Peak [Tmax]) as well as their combinations on the prognostic prediction of Acute Ischemic Stroke (AIS). Our findings aim to assist clinicians in making informed treatment decisions, developing personalized treatment plans for patients, and providing new ideas for clinical research. The main contributions of the research fall in the following three key areas.

1. Six groups of radiomics features of DSC-PWI images at different key time points were used to construct different prediction models. The impact of time point selection on the prediction effect was compared. Ultimately, it was confirmed that the selection of radiomics features of DSC-PWI sequences at three key time points has better prediction performance, which can reduce the computational complexity while ensuring accuracy.
2. To explore the performance of the four perfusion parameters (CBV, CBF, MTT, and Tmax) obtained by post-processing on the prediction aspect of AIS results, the prediction performance of a single parameter and the prediction performance of the combination of the four parameters were explicitly analyzed. The results confirmed that combining these parameters can effectively improve prediction accuracy.
3. The best prediction model was constructed by integrating source features derived from DSC-PWI sequences at three key time points and parameter features obtained from four perfusion parameter maps. The approach confirmed that the information on DSC-PWI sequences and perfusion parameters was complementary, highlighting the significant prognostic value inherent in the DSC-PWI sequences.

## 2 Materials

**Figure 1:**
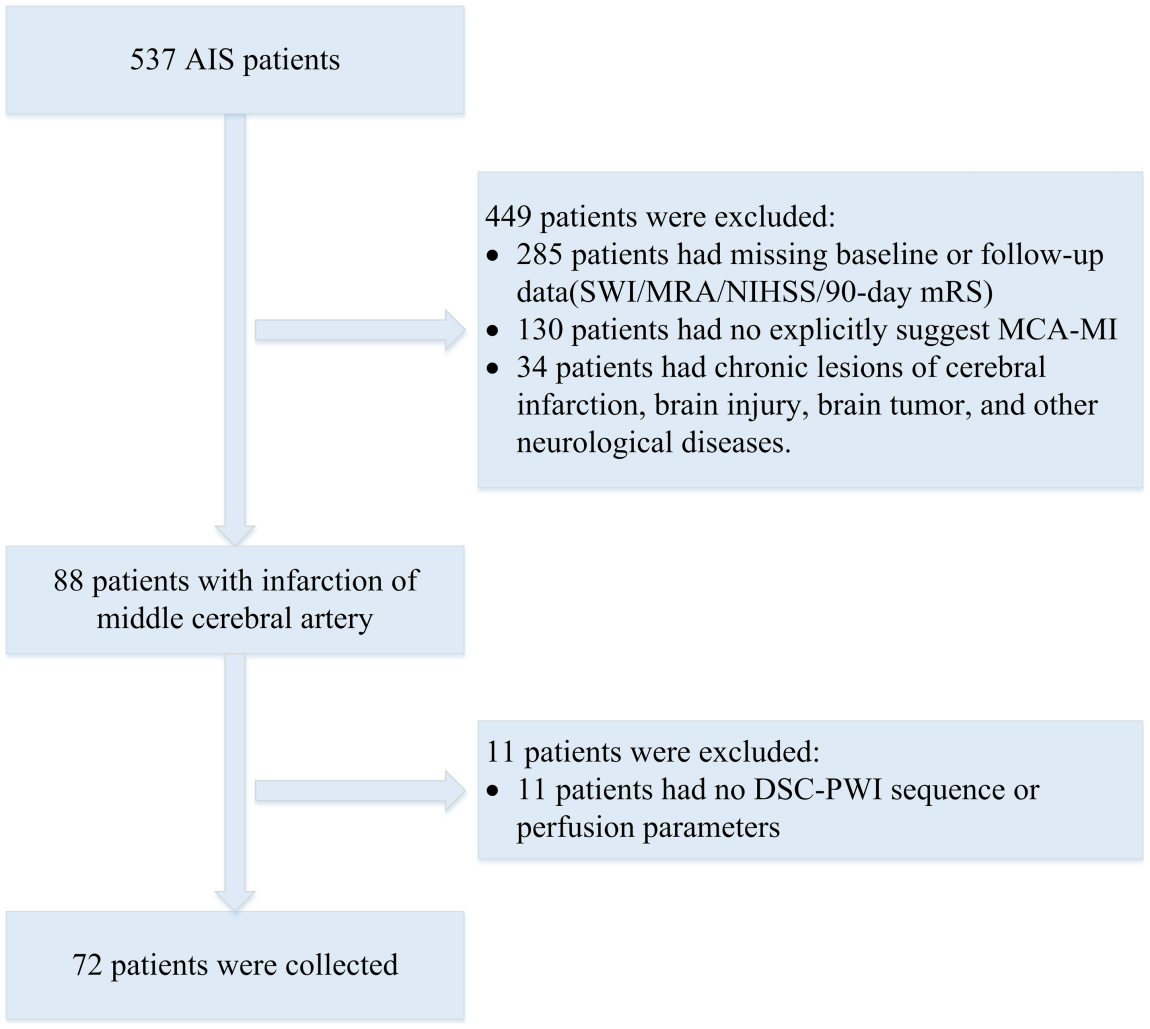
Flowchart of exclusion and inclusion of patients in our study. SWI, susceptibility weighted imaging; MRA, magnetic resonance angiography; NIHSS, National Institute of Health stroke scale.

The dataset for this study was provided by the neurology department of the Shanghai Fourth People’s Hospital, affiliated with the Tongji University School of Medicine, China. The dataset was retrospectively analyzed and included DSC-PWI images of 537 AIS patients from 2013 to 2019. All DSC-PWI sequences were approved by the Hospital Ethics Committee for ethical certification. For MR perfusion imaging, the contrast agent Gd-DTPA (Gadopentetate Dimeglumine, Shanghai Pharmaceutical Company, China) was infused intravenously at a rate of 4 mL/s at a dose of 0.2 mmol/kg according to the patient’s body weight, and a saline flush of 30 mL was given at the same flow rate. The patient’s inclusion and exclusion criteria were as follows (Figure 1): (1) The MR examinations were conducted within 24 h of symptom onset; (2) the presence of the middle cerebral artery (M1 segment) occlusion; (3) availability of complete clinical report including mRS score; (4) and availability of complete MR imaging sequences (DSC-PWI, CBV, CBF, MTT, and Tmax). A total of 72 AIS patients’ DSC-PWI images were selected for this study. Based on the 90-day mRS score obtained through telephone follow-up, the patients were categorized into two groups: 39 with a good prognosis (mRS ≤ 2) and 33 with a poor prognosis (mRS > 2).

Further statistical information of the patients and the scanning parameters of the DSC-PWI sequence are provided in Table 1. All DSC-PWI sequences were scanned on a 1.5-Tesla MR scanner (Siemens, Munich, Germany). The matrix size of each scan was 256 × 256, the number of slices was 19 or 20, the slice thickness was 5 mm, the slice spacing was 6.5 mm, the echo time (TE) was set to 32 ms, and the repetition time (TR) was 1,590 ms. The pixel bandwidth was 1,347 Hz/pixel, and the field of view (FOV) was 230 × 230 square millimeters to capture medium-sized regions of interest. Each sequence had a temporal resolution of 1.59 s, with a total of 50 sequences acquired. The study included 72 patients with a mean age of (71.32 ± 10.26) years and 22.2% of the participants were female. The patients’ functional outcomes were measured using the 90-day modified Rankine Scale (mRS) with a Mean–Variance of 2.60 ± 2.37.

**Table 1:** Scanning parameters of DSC-PWI images and patient information.

Scanning parameters of DSC-PWI images		Patient information	
Matrix	256 × 256	Patients	72
Number of slices	19 ± 1	Female (%)	16 (22.2%)
Spacing between slices	6.5 mm	Age (Mean ± Std)	71.32 ± 10.26
Number of measurements	50	90-day mRS (Mean ± Std)	2.60 ± 2.37
Thickness	5 mm	Onset time (Mean ± Std)	5.24 ± 4.15
TE/TR	32/1,590 ms	Hypertension	54
Pixel bandwidth	1,347 Hz/pixel	Diabetes	22
FOV	230 × 230 mm^2^	Atrial fibrillation	25
Temporal resolution	1.59 s	rt-PA therapy	19
		Thrombectomy	11
		rt-PA + Thrombolytic	10
		rt-PA + intraarterial stent	1

Among the 72 patients, 54 had hypertension, 22 had diabetes, and 25 had atrial fibrillation. Reperfusion of the ischemic penumbra was primarily achieved through thrombolysis and thrombectomy. The thrombolytic agent used was recombinant tissue plasminogen activator (rt-PA). A total of 30 patients underwent thrombolysis, and 21 patients underwent thrombectomy. Specifically:

- 12 patients received intravenous rt-PA (IV rt-PA).
- 3 patients received intraarterial rt-PA (IA rt-PA).
- 4 patients received both IV rt-PA and IA rt-PA.
- 11 patients underwent thrombectomy.
- 6 patients received IV rt-PA and thrombectomy.
- 2 patients received IA rt-PA and thrombectomy.
- 2 patients received IV rt-PA, IA rt-PA, and thrombectomy.
- 1 patient received IV rt-PA and intraarterial stenting.

## 3 Methods

**Figure 2:**
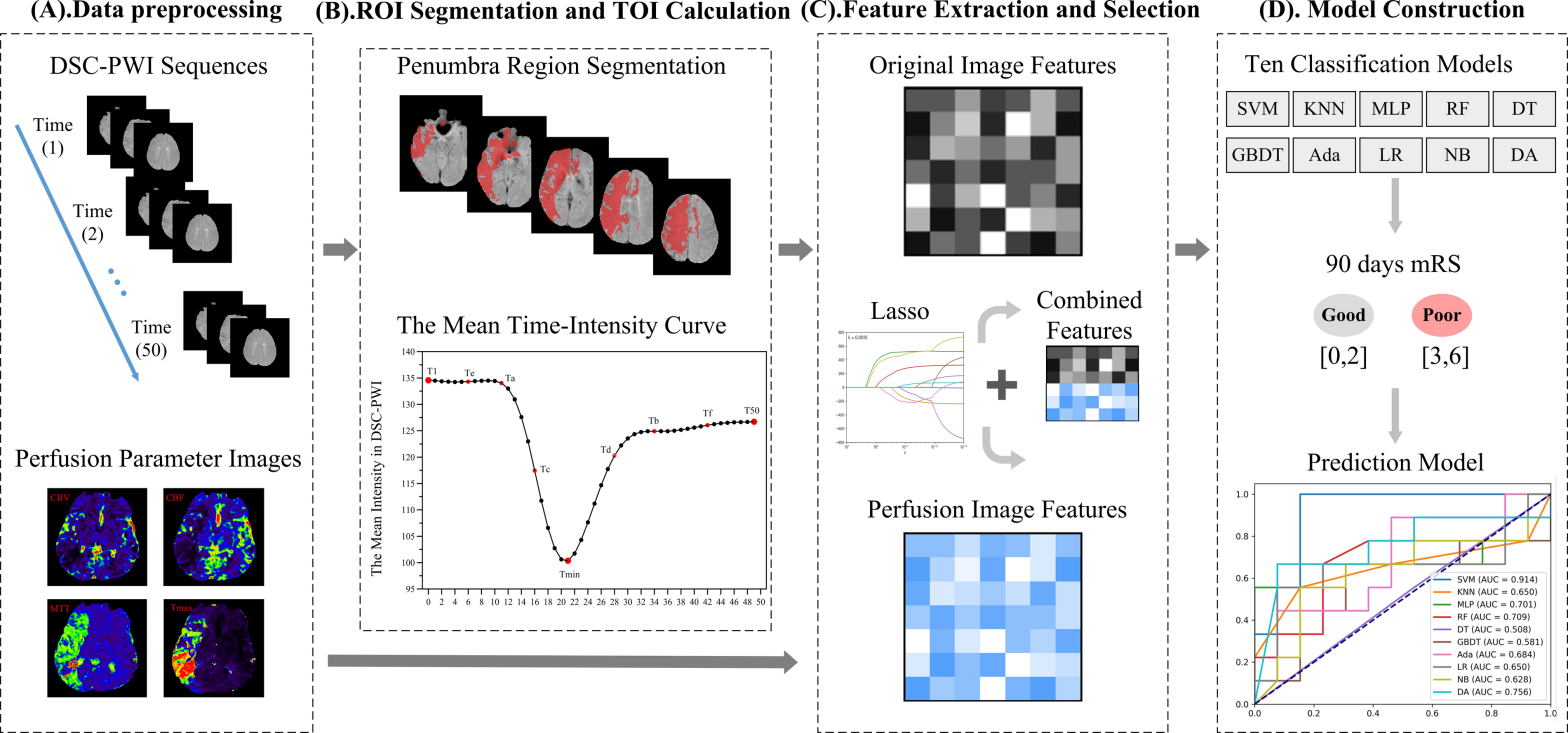
The framework of our method. **(A)** Data preprocessing. **(B)** Region of interest (ROI) segmentation and time of interest (TOI) calculation. **(C)** Feature extraction and selection. **(D)** Model construction.

The proposed framework of our conducted research is depicted in Figure 2, which consists of four main parts: (1) Data preprocessing; (2) Region of interest (ROI) segmentation and time of interest (TOI) computation; (3) Feature extraction and selection; (4) Prognosis prediction model construction. The data processing includes cleaning and registration of the input data to ensure consistency and improve the dataset’s quality, further generating new parameters. The ROI phase is intended to identify the areas of the brain affected mainly by ischemia, known as regions of interest (ROI). Following this, key time points, referred to as times of interest (TOI), are selected, which are essential for understanding stroke progression. The feature extraction part extracts meaningful features from the imaging data, including DSC-PWI sequences and perfusion parameters like CBV, CBF, MTT, and TTP. Further, feature selection techniques are applied to retain the most relevant features, minimize dimensionality, and enhance model efficiency. A machine learning-based prediction model is constructed using the selected features in the prognosis prediction model construction part. The proposed model is trained to predict patient outcomes, helping to guide clinical decision-making. The overall framework of our study is shown in Figure 2. Each section of the proposed framework has been described in detail.

### 3.1 Data preprocessing

**Figure 3:**
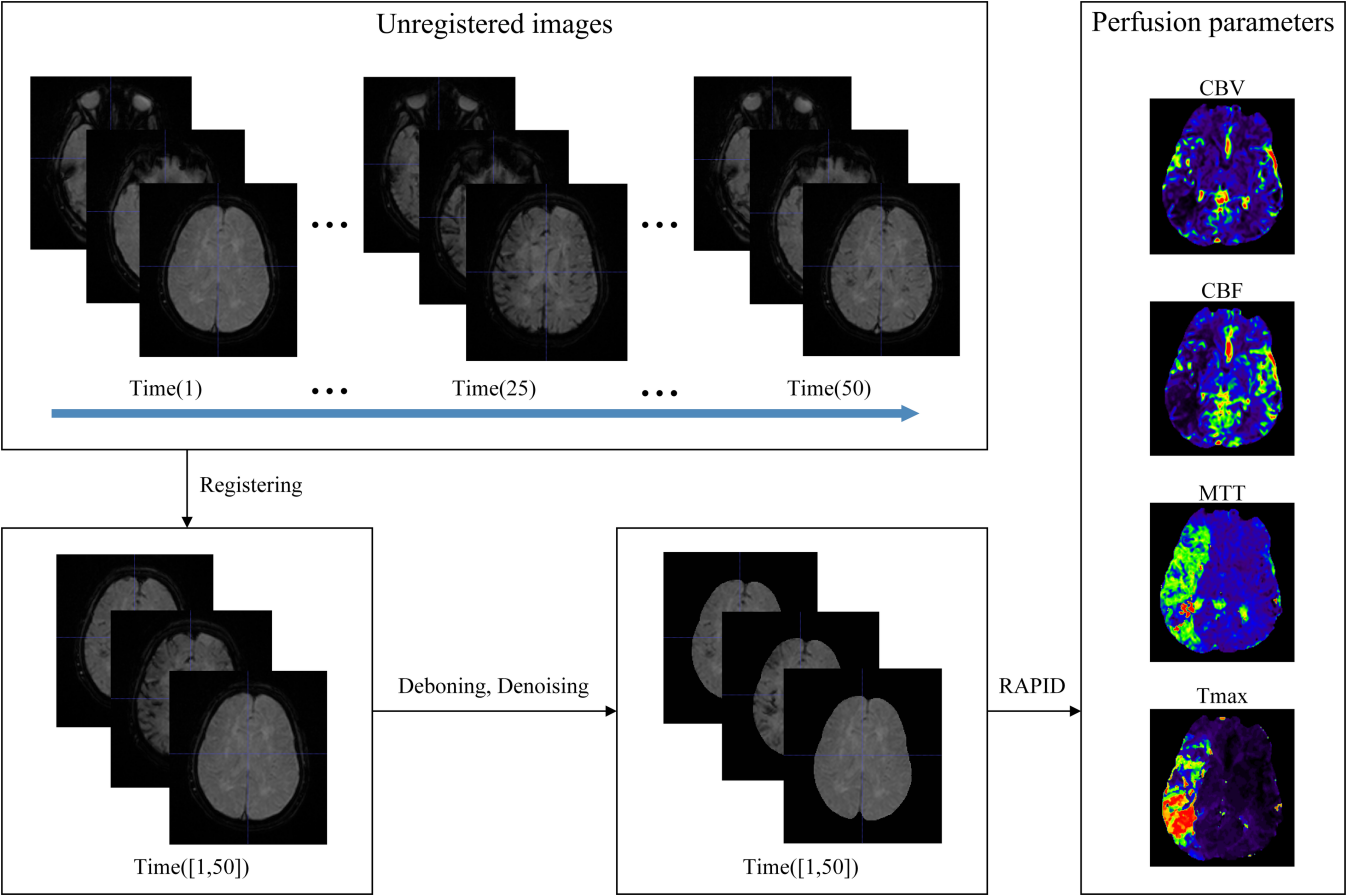
Data preprocessing. The unregistered multi-temporal DSC-PWI was registered, deboned, and denoised to generate perfusion parameters by specialized software.

The data preprocessing in this study mainly involved several key steps: registration of position, segmenting brain tissue regions, denoising of images, and generation of perfusion parameters, as outlined in Figure 3. First, the spatial position of the multi-temporal DSC-PWI sequences was registered using the neuroimaging software package FSL (18). This step was taken to eliminate the positional deviation caused by head movement that might exist during scanning. At the same time, non-brain tissues were removed using the BET method in FSL, and brain tissue regions were preserved for further feature parameter analysis. Then, the DSC-PWI sequences were denoised by processing the DSC-PWI sequences using three-shift panning with a window of 3 × 3 and a step size of 1 to improve the data quality. Finally, four perfusion parameter maps, CBV, CBF, MTT, and Tmax, were generated by back-convolution of the arterial input function. This process was fully automated using the RAPID Perfusion and Diffusion Processing software (19), which calculated the perfusion parameters directly from the DSC-PWI sequences.

### 3.2 ROI segmentation and TOI calculation

DSC-PWI enables the visualization of blood flow within brain tissue by capturing the perfusion process, making it suitable for identifying areas of ischemic penumbra and infarct core (20, 21). The threshold of the quantitative perfusion parameter Tmax > 6 s obtained through DSC-PWI post-processing is commonly used to identify the ischemic penumbra region (22–24). This has emerged as a novel strategy to identify patients most likely to benefit from treatment by targeting salvageable penumbra tissue (25). Therefore, in this study, the ischemic penumbra region was used as the ROI for feature extraction, and the segmentation of ROI was performed using the commercial software RAPID (19).

**Figure 4:**
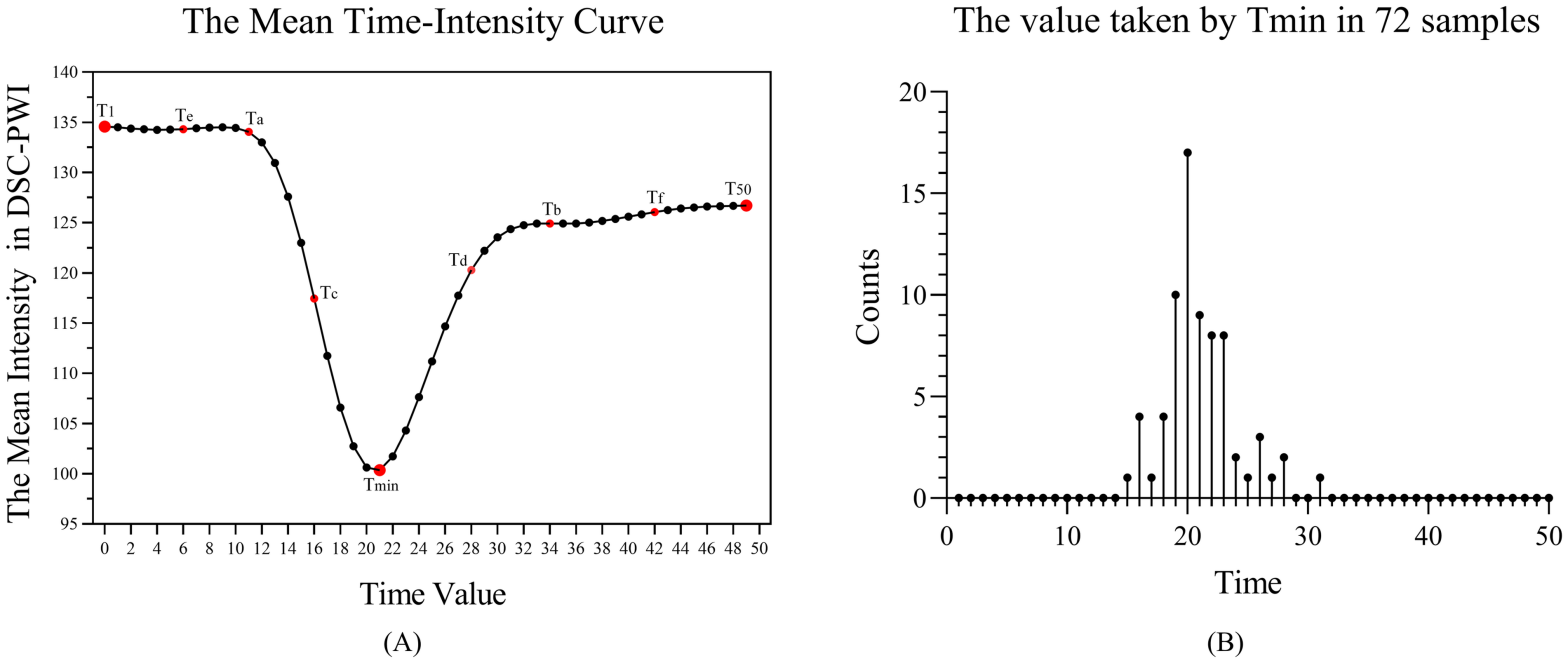
**(A)** Mean-time intensity curve of brain tissue at 50-time points of the DSC-PWI sequence. **(B)** The number of samples at 50-time points for the time value taken by Tmin, where Tmin is the time with the smallest mean intensity among the 50 times of the DSC-PWI sequence.

DSC-PWI images usually contain dozens of time sequences, extracting features for each sequence increases computational complexity. Thus, to avoid repeated extraction of anatomical information from the images while effectively retaining temporal information about the dynamic changes in blood flow, we chose DSC-PWI sequences at specific scanning time points to obtain the information. DSC-PWI reflects intracranial blood flow status by inducing changes in tissue signal intensity through the contrast agent. When the contrast agent reaches poorly perfused brain tissues, it is reflected in the DSC-PWI sequence as a small or unchanged signal intensity value (26). By analyzing the signal intensity within the brain tissue over time in the DSC-PWI sequence following contrast injection, Figure 4A shows the mean time-intensity curve for all pixel points within the brain tissue of a particular patient, measured across 50 time points. This curve illustrates a distinct pattern of decreasing and increasing signal intensity in the brain tissue.

To consider the influence of the time-dimensional information and the number of selected time points on the prediction results, we identified nine key time points, referred to as Times of Interest (TOI). These include the first moment (T1), the moment with the lowest average intensity (Tmin), the last moment (T50), the midpoint between T1 and Tmin (Ta), between Tmin and T50 (Tb), between Ta and Tmin (Tc), between Tmin and Tb (Td), between Ta and T1 (Te), and between Tb and T50 (Tf). Figure 4B shows the number distribution of samples at 50-time points for the time values taken for Tmin. We found that the Tmin of the 72 samples is mainly concentrated at time points 19–23, with the largest number of samples taken at the 20th time point.

Eventually, five experimental groups were formed based on nine selected TOIs, namely Tmin, T1 + Tmin + T50, T1 + Ta + Tmin + Tb + T50, T1 + Ta + Tc + Tmin + Td + Tb + T50, and T1 + Te + Ta + Tc + Tmin + Td + Tb + Tf + T50. The DSC-PWI sequences for these five groups were labeled as 1PWI, 3PWI, 5PWI, 7PWI, and 9PWI, respectively. We will mainly focus on extracting the cerebral blood flow features associated with changes in the cerebral blood flow states from the DSC-PWI sequences at these selected time points and explore the associations of these features with the prediction of short-term prognosis of AIS. In addition, to investigate whether features from all scanned time points are essential, we also extracted features for all 50 moments of DSC-PWI sequences (50PWI) for prognostic prediction.

### 3.3 Feature extraction and selection

#### 3.3.1 Radiomics features extraction of DSC-PWI sequences at critical time points and perfusion parameter maps

We used the Pyradiomics toolkit on Python 3.7 to extract radiomics features from ROIs (27). The extracted feature classes were divided into six classes: (1) 18 First-order statistics features (First-order); (2) 24 Gray Level Co-occurrence Matrix features (GLCM); (3) 16 Gray Level Run Length Matrix features (GLRLM); (4) 16 Gray Level Size Zone Matrix features (GLSZM); (5) 5 Neighboring Gray Tone Difference Matrix features (NGTDM); and (6) 14 Gray Level Dependence Matrix features (GLDM). Table 2 provides the specific feature terms associated with each class.

**Table 2:** A summary of the high-throughput radiomics features extracted.

Feature classes	Feature names
First_order	10Percentile, 90Percentile, Energy, Entropy, Interquartile Range, Kurtosis, Maximum, Mean Absolute Deviation, Mean, Median, Minimum, Range, Robust Mean Absolute Deviation, Root Mean Squared, Skewness, Total Energy, Uniformity, Variance
GLCM	Autocorrelation, Joint Average, Cluster Prominence, Cluster Shade, Cluster Tendency, Contrast, Correlation, Difference Average, Difference Entropy, Difference Variance, Joint Energy, Joint Entropy, Informational Measure of Correlation 1, Informational Measure of Correlation 2, Inverse Difference Moment, Maximal Correlation Coefficient, Inverse Difference Moment Normalized, Inverse Difference, Inverse Difference Normalized, Inverse Variance, Maximum Probability, Sum Average, Sum Entropy, Sum of Squares
GLRLM	Short Run Emphasis, Long Run Emphasis, Gray Level Non-Uniformity, Gray Level Non-Uniformity Normalized, Run Length Non-Uniformity, Run Length Non-Uniformity Normalized, Run Percentage, Gray Level Variance, Run Variance, Run Entropy, Low Gray Level Run Emphasis, High Gray Level Run Emphasis, Short Run Low Gray Level Emphasis, Short Run High Gray Level Emphasis, Long Run Low Gray Level Emphasis, Long Run High Gray Level Emphasis
GLSZM	Small Area Emphasis, Large Area Emphasis, Gray Level Non-Uniformity, Gray Level Non-Uniformity Normalized, Size-Zone Non-Uniformity, Size-Zone Non-Uniformity Normalized, Zone Percentage, Gray Level Variance, Zone Variance, Zone Entropy, Low Gray Level Zone Emphasis, High Gray Level Zone Emphasis, Small Area Low Gray Level Emphasis, Small Area High Gray Level Emphasis, Large Area Low Gray Level Emphasis, Large Area High Gray Level Emphasis
NGTDM	Coarseness, Contrast, Busyness, Complexity, Strength
GLDM	Small Dependence Emphasis, Large Dependence Emphasis, Gray Level Non-Uniformity, Dependence Non-Uniformity, Dependence Non-Uniformity Normalized, Gray Level Variance, Dependence Variance, Dependence Entropy, Low Gray Level Emphasis, High Gray Level Emphasis, Small Dependence Low Gray Level Emphasis, Small Dependence High Gray Level Emphasis, Large Dependence Low Gray Level Emphasis, Large Dependence High Gray Level Emphasis

To capture the feature information of the image in different frequency domains, we used six filters to transform the image type, supplementing the six classes of feature information extracted from the original image. These filters included Laplacian of Gaussian (Log) with the sigma values {1.0, 2.0, 3.0, 4.0, 5.0}, as well as square, square root, logarithmic, exponential, and eight combinations of wavelet transform in three dimensions generated by high-pass and low-pass filters (LLH, LHL, LHH, HLL, HLH, HHL, HHH, LLL). So, for each image, we extracted 1,674 grouped features calculated as (18 × (18 + 24 + 16 + 16 + 5 + 14) = 1,674). Since the perfusion parameters CBV, CBF, MTT, and Tmax were generated by post-processing the DSC-PWI sequences, we refer to the features obtained from the six DSC-PWI sequences as the source features and denote them as 1PWI_F, 3PWI_F, 5PWI_F, 7PWI_F, 9PWI_F, and 50PWI_F. Features derived from the CBV, CBF, MTT, and Tmax are parametric features labeled as CBV_F, CBF_F, MTT_F, and Tmax_F, respectively. In addition, the radiomics feature is named according to its source image, filter type, feature class, and the specific feature name connected by underscores, such as “CBV_ logarithm_firstorder_Kurtosis,” which denotes the Kurtosis radiomics feature in the first_order class extracted from the CBV that passed through the logarithm filter.

#### 3.3.2 Radiomics features selection and combination

Since radiomics features extracted from images are diverse and have different scales, these different scale features have been assigned different weights in the feature selection and classification process, thus affecting the outcomes (28, 29). Research has also shown that the model constructed with normalized features has better prediction performance than the model built without normalized features (30). Therefore, in this study, after extracting radiomics features from the DSC-PWI sequences and perfusion parameters, we first applied mean normalization on the features to achieve the compression of all feature terms in the interval [−1, 1].

Then, since we extracted features from multiple DSC-PWI sequences with the same anatomical structure, redundant information and increased noise can arise. To address this, we followed the approach of many studies by using a combination of T-test and Least Absolute Shrinkage and Selection Operator (Lasso) for feature selection (31–33). The T-test is used to select and retain the significant features with *p* < 0.05 that can significantly differentiate between the two categories, achieving an initial dimensionality reduction of the features. The goal was to minimize the influence of redundant features on the Lasso algorithm, thereby reducing the risk of unstable selection results and also lowering computational complexity and the risk of overfitting for Lasso select features. The Lasso algorithm, commonly employed to identify relevant features for robust classification models (34). However, in high-dimensional datasets where the number of features far exceeds the number of samples, traditional Lasso regression may fail to effectively identify the truly important features, leading to false positive features. To overcome this, we adopted a threshold Lasso algorithm (35), which adds an extra thresholding step after Lasso’s L1 regularization. The purpose is to set a threshold after Lasso regression has provided the coefficient estimates, further removing features with small coefficients that contribute little to the prediction, thus improving the model’s accuracy and stability. We retained features with weight coefficients greater than 0.02, which are more predictive for the target task category. This method helps by “thresholding” non-significant coefficients to zero, effectively removing unnecessary features and reducing noise interference.

Specifically, we first extracted all six categories of radiomics features from the entire dataset, then normalized the data and divided it into a training set and a validation set. Next, we applied the T-test and Lasso algorithm sequentially to the features in the training set to select the relevant features, which were then used to construct the prognostic model. Thus, we constructed six source feature models with six source features (1PWI_F, 3PWI_F, 5PWI_F, 7PWI_F, 9PWI_F, and 50PWI_F) and four parametric feature models with four perfusion parameter features (CBV_F, CBF_F, MTT_F, and Tmax_F). Finally, selected relevant feature terms were identified in all radiomics of the validation set and used in the constructed prognostic model for outcome prediction of the validation set data.

Since different perfusion parameter maps characterize different hemodynamic information, so relying on a single parameter alone would lead to incomplete information. To address this, we combined the relevant features obtained from the four sets of parameter maps and noted the perfusion parameter combination feature as PerfusionF. In addition, the complementary nature of critical time-point DSC-PWI images, which provide visual image information about the cerebrovascular perfusion situation and anatomical structures, alongside the perfusion parameter maps, which provide detailed, quantitative hemodynamic information. Thus, we combined the obtained source features and PerfusionF to construct a thoroughly combined feature (CombinedF) model that can accurately predict good and poor prognosis in AIS patients. This CombinedF model is also the primary recommendation in our study.

### 3.4 Prognosis prediction model construction

**Figure 5:**
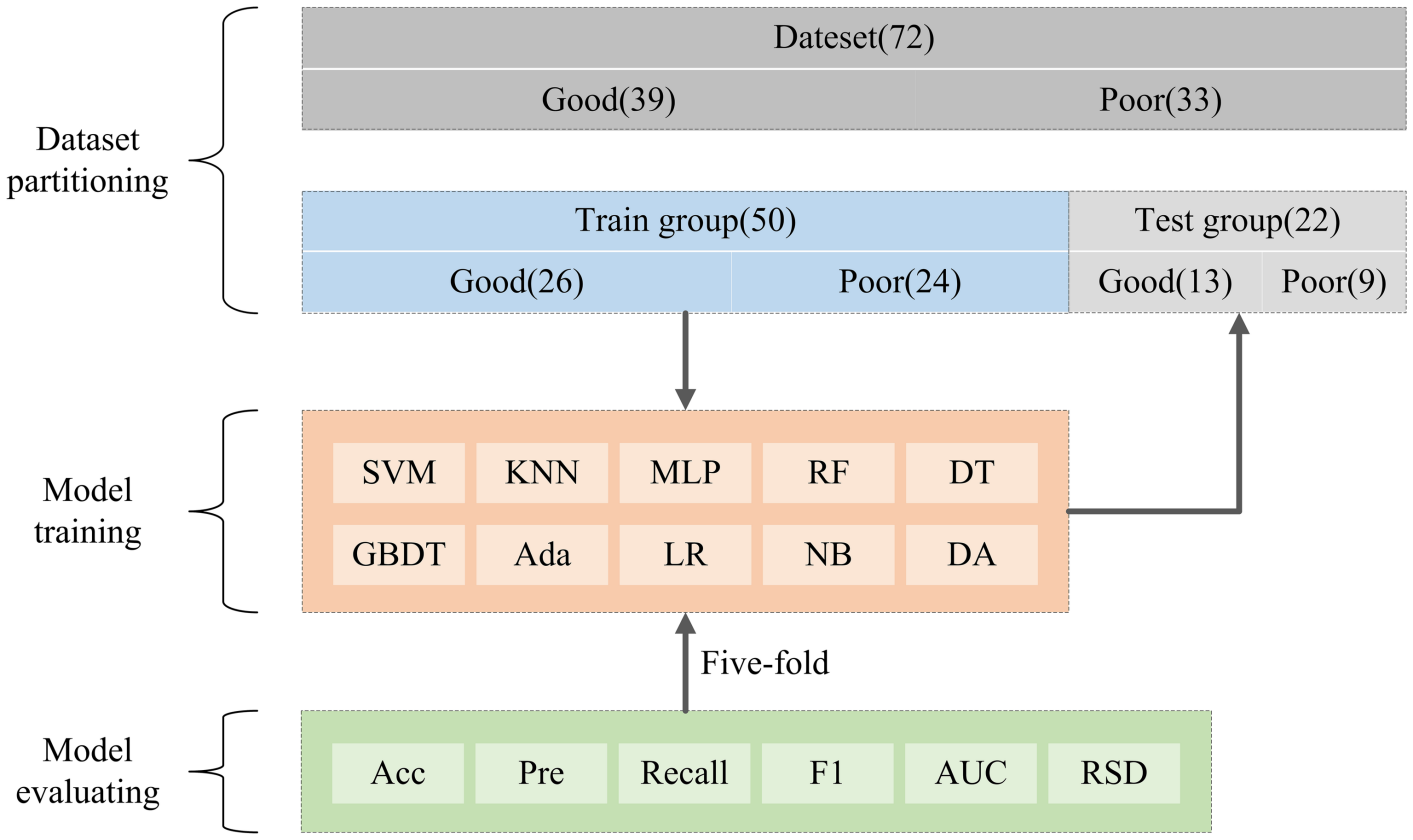
The data partitioning process and model training.

The dataset split and model training process is shown in Figure 5. A total of 72 cases were collected in this study, which were categorized into 39 cases with good prognosis and 33 cases with poor prognosis based on the mRS score. Prior to model training, we used the train_test_split() function to divide the dataset into a training group (*n* = 50) and a validation group (*n* = 22) with a 7:3 ratio. Additionally, the StratifiedKFold (n_splits = K) function has been employed, where *K* = 5, to perform a five-fold cross-validation for evaluating the training model. Finally, the trained model was tested on an independent validation set to obtain the final classification results.

Considering that the performance of the same features can differ across various models, to select models that are more sensitive to the radiomics features of this study and more effective for the classification task, we constructed the prognostic models by using 10 ML models with different classification principles, precisely: Support Vector Machines (SVM), K-nearest Neighbor (KNN), Multi-layer perceptual neural networks (MLP), Random forest (RF), Decision Tree (DT), Gradient Boosting Decision Tree (GBDT), Adaptive boosting (Ada), Logistic Regression (LR), Gaussian NB (NB), and Discriminant Analysis (DA).

All the predictive models were trained in the same training cohort and tested in the same validation cohort. The classification performance of the prediction models was evaluated by five classification metrics, including Accuracy (Acc), Precision (Pre), Recall, F1-score (F1), and Area Under the Curve (AUC). The Area Under the Curve (AUC) is derived from the Receiver Operating Characteristic (ROC) curve and serves as the primary metric for assessing the predictive performance in this study. Moreover, the model’s stability is quantified by the coefficient of variation of the AUC values from the training set, referred to as the Relative Standard Deviation (RSD) (36). Equation 1 presents the RSD formula, where a lower RSD indicates a more stable model.


(1) $$RSD=\frac{S_{AUC}}{\bar{AUC}}\times 100\%$$

where S_AUC_ denotes the standard deviation of the AUC value and $\bar{AUC}$ denotes the mean of five-fold cross-validated AUC value.

### 3.5 Comparative experimental design

#### 3.5.1 Comparative experiments based on six sets of source features

**Figure 6:**
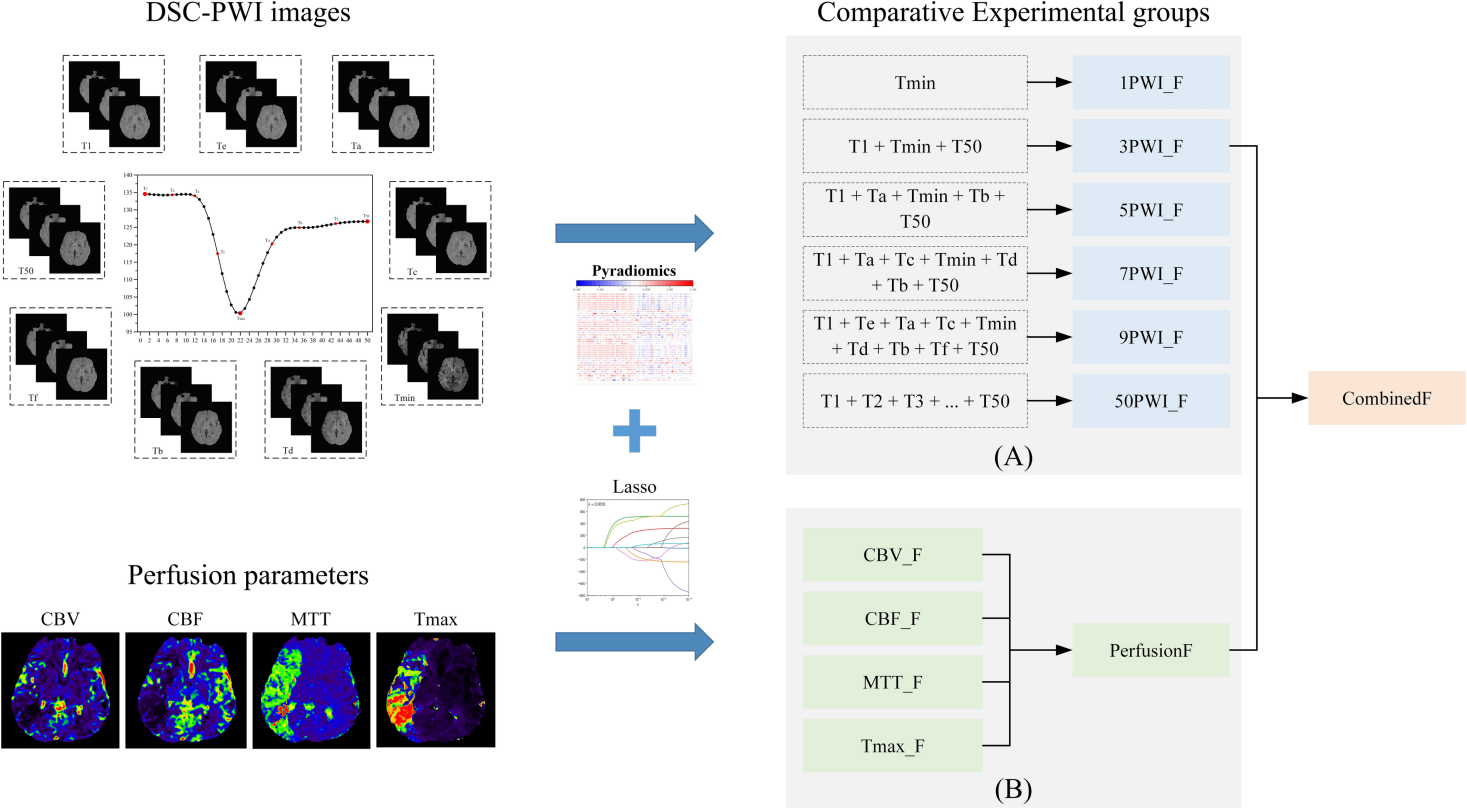
The overall experimental framework in this study consists of **(A)** comparative experimental groups based on six sets of source features and **(B)** comparative experimental groups based on four sets of parametric features and parameter combined features.

DSC-PWI enables real-time monitoring of the perfusion status of brain tissue by rapidly acquiring multiple sequences of images regarding changes in the contrast agent. We identify three crucial time points by analyzing the mean time-intensity curve in Figure 4A. The curve’s starting point indicates the basal signal intensity without the contrast agent; the lowest point of the curve suggests the peak of the contrast agent concentration, which reflects the maximum concentration of the contrast agent within the blood flow channel. The endpoint indicates the intensity of the contrast agent remaining after the contrast agent is washed out of the blood vessel and partially absorbed within the tissue. This endpoint reflects the vascular clearance of the blood flow and the cellular metabolism ability within the tissue. Consequently, the prediction model based on DSC-PWI sequence features of these three key time points is a recommended method in this study. Moreover, different time points were selected for comparison experiments to validate the advantages of these three critical times, as illustrated in Figure 6A.

#### 3.5.2 Comparative experiments based on four sets of single-parameter features and one set of parameters combined feature

CBV indicates the cerebral blood volume of a certain amount of brain tissue, which can reflect the expansion and contraction of blood vessels. CBF measures the volume of blood passing through a given tissue per unit of time, indicating local vascular resistance. MTT is the average duration for a contrast agent to move through brain tissue, while Tmax denotes the time to peak contrast agent concentration. Prolonged MTT and Tmax both can reflect delayed blood flow or vascular obstruction. These four perfusion parameters contain different information and have important clinical implications in diagnosing and treating ischemic stroke. To explore the most relevant parameters among these four parameters to AIS prognosis, four single-parameter prediction models were developed for comparison to determine the predictive ability of different perfusion parameters in AIS prognosis. In addition, since the four parameters may contain complementary information relevant to AIS prognostic prediction, we also combined the four single-parameter features. We assessed their predictive capability against that of the individual parameters, as illustrated in Figure 6B.

## 4 Results

### 4.1 Selected radiomics features

**Figure 7:**
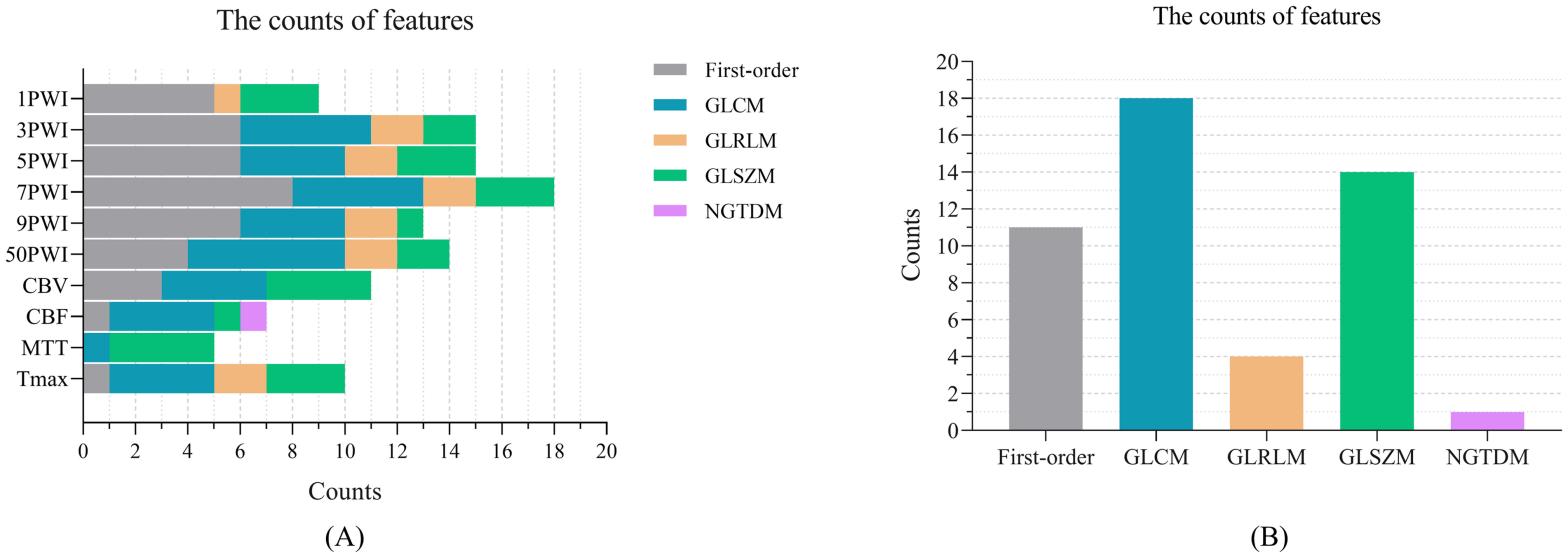
The number of features selected across different feature categories: **(A)** The relevant features selected by Lasso in the 10 feature groups, organized by feature category, and **(B)** the features within the CombinedF group, categorized by feature type.

We applied the T-test and Lasso algorithm to select relevant features, finally identifying 9, 15, 15, 18, 13, and 14 features from the six groups of source features (1PWI_F, 3PWI_F, 5PWI_F, 7PWI_F, 9PWI_F, and 50PWI_F) and 11, 7, 5, and 10 relevant features from four groups of perfusion parameter features (CBV_F, CBF_F, MTT_F, and Tmax_F), respectively. We then compared the performance of the predictive models constructed from the six groups of source features and selected the best-performing set, combined with the perfusion parameter combined feature (PerfusionF) to create a fully combined feature set (CombinedF) consisting of 48 radiomics features. Figure 7 depicts the number of features across different feature classes, revealing that GLDM features were excluded from all sequences, and only one NGTDM feature, “logarithm_ngtdm_Contrast,” was selected in the CBF.

### 4.2 Performance of CombinedF prediction model

In this study, radiomics features of DSC-PWI sequences at three key time points were combined with radiomics features of perfusion parameters generated by their post-processing. This combined feature (CombinedF) set was used to train 10 ML models, followed by testing the classification performance of these models on a validation cohort for predicting AIS prognostic outcomes. The results are summarized in Table 3, indicating the SVM-based model has the best predictive ability, with AUC, Acc, Pre, Recall, and F1 of 0.915, 0.818, 0.778, 0.778, and 0.778, respectively, and the RSD of the model is <6%, underscoring its robustness. In addition, the DA model performed well with an AUC, Acc, Pre, Recall, and F1 of 0.846, 0.818, 0.692, 1.000, and 0.818, respectively, and the RSD < 9%. We found that on RF, DT, GBDT, and Ada models, the RSD of the training process is >10%, and the developed prediction models are not stable enough. Among the 10 models tested, SVM and DA models outperformed the others, which shows that the performance of the constructed prediction models may differ depending on the classifiers chosen.

**Table 3:** Performance of CombinedF prediction model.

	Classifier	Train_RSD↓	Test_AUC↑	Test_Acc↑	Test_Pre↑	Test_Recall↑	Test_F1↑
CombinedF model	SVM	0.055	0.915	0.818	0.778	0.778	0.778
	KNN	0.056	0.714	0.773	0.750	0.667	0.706
	MLP	0.023	0.726	0.818	0.857	0.667	0.750
	RF	0.194	0.731	0.682	0.667	0.444	0.533
	DT	0.123	0.564	0.545	0.462	0.667	0.545
	GBDT	0.235	0.556	0.500	0.417	0.556	0.476
	Ada	0.125	0.684	0.591	0.500	0.556	0.526
	LR	0.031	0.684	0.682	0.625	0.556	0.588
	NB	0.038	0.662	0.636	0.556	0.556	0.556
	DA	0.082	0.846	0.818	0.692	1.000	0.818
	Mean ± Std	0.096 ± 0.072	0.708 ± 0.110	0.686 ± 0.118	0.630 ± 0.145	0.645 ± 0.155	0.628 ± 0.123

Furthermore, to investigate whether the clinical baseline data of enrolled patients had a potential impact on the model, we analyzed the statistical differences between these baseline variables and the two prognostic groups (good and bad prognostic). The analyzed variables included sex, age, hypertension, diabetes, atrial fibrillation, and onset time. Among them, sex, hypertension, diabetes, and atrial fibrillation were categorical variables, for which we performed chi-square tests to assess statistical differences between the two prognostic groups. Age and onset time were continuous variables, for which we conducted independent sample t-tests to evaluate statistical differences. The results of our analysis are presented in Table 4. Except for diabetes, no other baseline clinical variables showed significant differences. Given the small sample size, the statistical significance of diabetes may be due to random factors. Therefore, its potential impact on the predictive model can be disregarded.

**Table 4:** Results of the statistical difference analysis between the clinical baseline data and the two prognostic groups.

Clinical datas	Good prognosis (*n* = 39)	Bad prognosis (*n* = 33)	*p*-value
Sex	7 (Female)	9 (Female)	0.343
Hypertension	30	24	0.682
Diabetes	7	15	0.032*
Atrial fibrillation	14	11	0.820
Age	69.69 ± 9.06	73.24 ± 11.22	0.157
Onset time	5.58 ± 4.96	4.80 ± 2.76	0.373

*p-value < 0.05.

### 4.3 Performance of comparative experimental groups

#### 4.3.1 Performance of six sets of source features

Six groups of source features (1PWI_F, 3PWI_F, 5PWI_F, 7PWI_F, 9PWI_F, and 50PWI_F) at different time points were used to construct prediction models, and the performance of models was assessed using six evaluation metrics. Table 5 shows the top three models (MLP, LR, and DA) and the Mean–Variance for all 10 ML models. Performance ranking for the models using these feature groups is as follows: 3PWI > 9PWI > 5PWI > 50PWI > 7PWI > 1PWI, among which the 3PWI feature group performs the best on the MLP model, with the AUC, Acc, Pre, Recall, and F1 of 0.863, 0.818, 0.778, 0.778, and 0.778, respectively, and the RSD < 10%. It could be noted that the prediction results of the model developed by the 50PWI feature group are not the best, but the model constructed by it shows better stability overall. This stability may be brought about by the large amount of computational data. In the future, adding more cases will eliminate this discrepancy.

**Table 5:** Performance of prediction models constructed by six source feature groups.

Feature Group	Classifier	Train_RSD↓	Test_AUC↑	Test_Acc↑	Test_Pre↑	Test_Recall↑	Test_F1↑
1PWI_F	MLP	0.280	0.590	0.545	0.444	0.444	0.444
	LR	0.259	0.709	0.682	0.600	0.667	0.632
	DA	0.241	0.744	0.727	0.667	0.667	0.667
	Mean ± Std	0.245 ± 0.040	0.649 ± 0.078	0.609 ± 0.081	0.523 ± 0.088	0.589 ± 0.118	0.551 ± 0.093
3PWI_F	MLP	0.096	0.863	0.818	0.778	0.778	0.778
	LR	0.137	0.829	0.727	0.667	0.667	0.667
	DA	0.057	0.821	0.773	0.667	0.889	0.762
	Mean ± Std	0.166 ± 0.098	0.762 ± 0.134	0.714 ± 0.134	0.646 ± 0.133	0.756 ± 0.102	0.691 ± 0.107
5PWI_F	MLP	0.141	0.692	0.591	0.500	0.667	0.571
	LR	0.110	0.761	0.773	0.700	0.778	0.737
	DA	0.123	0.786	0.773	0.750	0.667	0.706
	Mean ± Std	0.149 ± 0.066	0.713 ± 0.074	0.673 ± 0.085	0.589 ± 0.103	0.667 ± 0.139	0.622 ± 0.112
7PWI_F	MLP	0.135	0.795	0.727	0.636	0.778	0.700
	LR	0.137	0.701	0.682	0.571	0.889	0.696
	DA	0.046	0.744	0.727	0.615	0.889	0.727
	Mean ± Std	0.138 ± 0.036	0.701 ± 0.121	0.645 ± 0.113	0.544 ± 0.135	0.622 ± 0.241	0.574 ± 0.178
9PWI_F	MLP	0.096	0.701	0.682	0.600	0.667	0.632
	LR	0.131	0.641	0.727	0.714	0.556	0.625
	DA	0.122	0.692	0.727	0.667	0.667	0.667
	Mean ± Std	0.126 ± 0.028	0.741 ± 0.077	0.718 ± 0.064	0.662 ± 0.073	0.667 ± 0.128	0.656 ± 0.077
50PWI_F	MLP	0.049	0.632	0.500	0.400	0.444	0.421
	LR	0.049	0.675	0.636	0.545	0.667	0.600
	DA	0.033	0.709	0.591	0.500	0.667	0.571
	Mean ± Std	0.100 ± 0.100	0.709 ± 0.043	0.627 ± 0.060	0.532 ± 0.061	0.689 ± 0.115	0.599 ± 0.079

#### 4.3.2 Performance of four sets of single-parameter features and one set of parameters combined feature

Four groups of single-parameter features (CBV_F, CBF_F, MTT_F, and Tmax_F) obtained with different perfusion parameters were used to develop prediction models, and their performance was evaluated across six metrics. Table 6 shows the scores of the best three models (MLP, NB, and DA) and the Mean–Variance of the 10 ML models. The results indicate that the MTT feature group performs significantly better than the other three. The order of the four groups is MTT > CBV > CBF > Tmax, in which the MTT feature group performs the best on the DA model, with the best AUC, Acc, Pre, Recall, and F1 of 0.821, 0.727, 0.714, 0.556, and 0.625, respectively, in which the prediction model constructed by CBV feature group showed more stable performance.

**Table 6:** Performance of predictive models constructed by four single-parameter feature groups.

Feature group	Classifier	Train_RSD↓	Test_AUC↑	Test_Acc↑	Test_Pre↑	Test_Recall↑	Test_F1↑
CBV_F	MLP	0.091	0.624	0.591	0.500	0.667	0.571
	NB	0.125	0.598	0.636	0.556	0.556	0.556
	DA	0.062	0.624	0.636	0.545	0.667	0.600
	Mean ± Std	0.143 ± 0.081	0.598 ± 0.062	0.586 ± 0.066	0.49 ± 0.075	0.533 ± 0.147	0.507 ± 0.105
CBF_F	MLP	0.222	0.667	0.727	0.667	0.667	0.667
	NB	0.210	0.615	0.636	0.556	0.556	0.556
	DA	0.173	0.607	0.545	0.455	0.556	0.500
	Mean ± Std	0.225 ± 0.033	0.622 ± 0.035	0.623 ± 0.071	0.537 ± 0.080	0.611 ± 0.095	0.570 ± 0.078
MTT_F	MLP	0.144	0.795	0.727	0.636	0.778	0.700
	NB	0.142	0.812	0.727	0.800	0.444	0.571
	DA	0.171	0.821	0.727	0.714	0.556	0.625
	Mean ± Std	0.162 ± 0.032	0.775 ± 0.042	0.704 ± 0.039	0.649 ± 0.083	0.689 ± 0.137	0.653 ± 0.039
Tmax_F	MLP	0.146	0.615	0.545	0.444	0.444	0.444
	NB	0.166	0.573	0.636	0.556	0.556	0.556
	DA	0.132	0.650	0.682	0.625	0.556	0.588
	Mean ± Std	0.153 ± 0.053	0.572 ± 0.079	0.577 ± 0.107	0.486 ± 0.144	0.433 ± 0.123	0.457 ± 0.130

**Figure 8:**
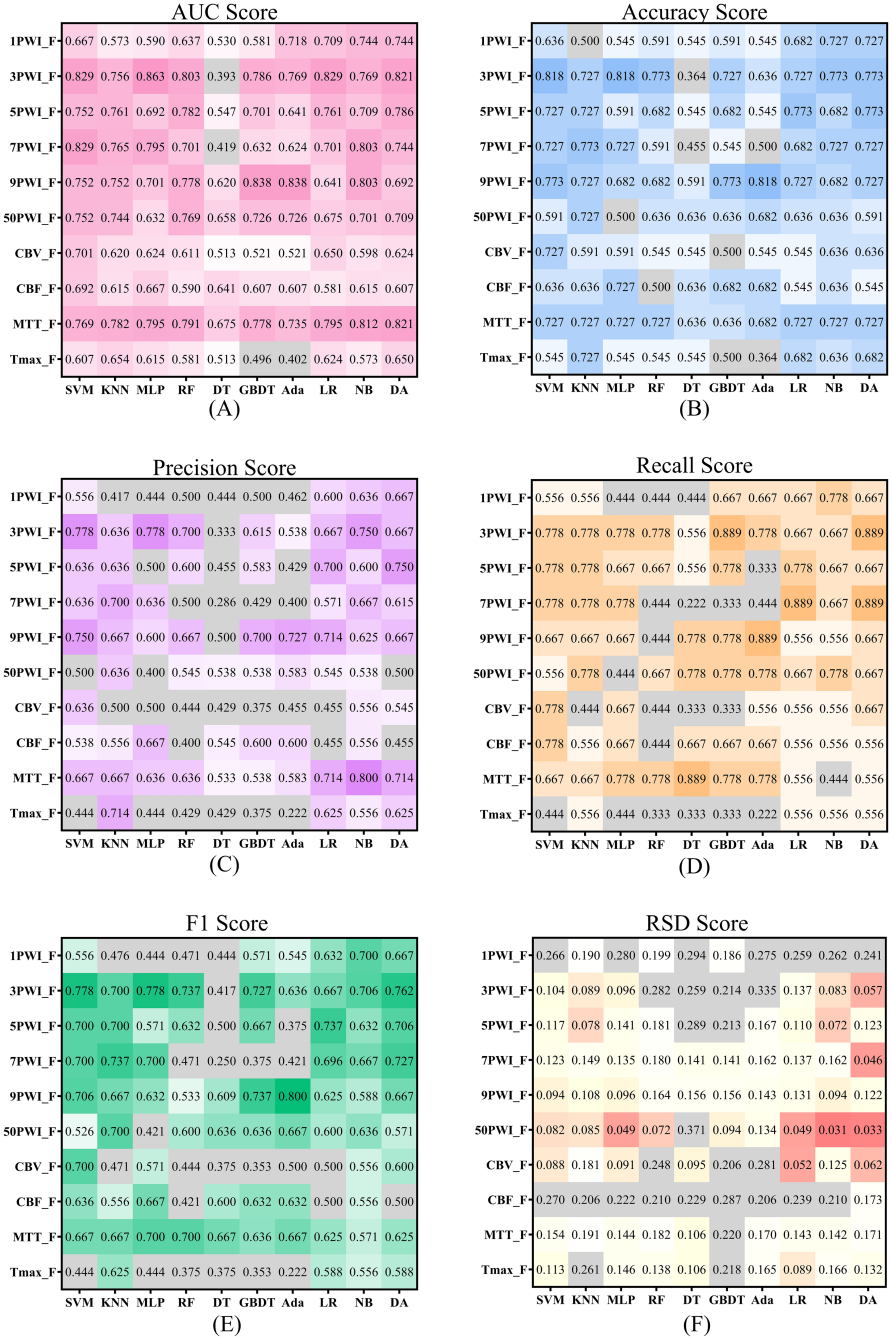
Scores for source and parameter features across six evaluation metrics are shown, with darker colors indicating more robust performance. Panels **(A–E)** use color to represent scores above 0.5, where darker shades signify higher scores. Panel **(F)** applies color to scores below 0.2 and red to those below 0.1, with darker hues highlighting better scores.

Considering the existence of complementary prognostic information contained in different single parameters, we developed a PerfusionF model by integrating features selected from four single-parameter features. The prediction performance of the PerfusionF model is detailed in Table 7. To compare the results between single-parameter features and the combined features, the results of the six metrics of the single-parameter features on the 10 ML models are illustrated in Figure 8. Analyzing Table 7 and Figure 8, we find that the PerfusionF model achieved the best performance on the SVM model. It surpassed the single-parameter features with AUC, Acc, Pre, Recall, and F1 of 0.889, 0.864, 0.800, 0.889, and 0.842. This represents an improvement over the CBV of 18.8, 13.7, 16.4, 11.1, and 14.2%. The improvement over the CBF is 19.7, 22.8, 26.2, 11.1, and 20.6%, respectively. Then, over the MTT, it is 12.0, 13.7, 13.3, 22.2, and 17.5%, respectively. Lastly, the improvement over the Tmax is 28.2, 31.9, 35.6, 44.5, and 39.8%, respectively. However, in some cases, the performance of the combined parametric features was lower than the single-parameter features on certain models. This inconsistency may be because of the inconsistent sensitivity of the different models to the features, resulting in a large difference in the final classification performance.

**Table 7:** Performance of PerfusionF prediction model.

	Classifier	Train_RSD↓	Test_AUC↑	Test_Acc↑	Test_Pre↑	Test_Recall↑	Test_F1↑
PerfusionF model	SVM	0.084	0.889	0.864	0.800	0.889	0.842
	KNN	0.082	0.611	0.682	0.600	0.667	0.632
	MLP	0.044	0.590	0.591	0.500	0.556	0.526
	RF	0.059	0.573	0.636	0.600	0.333	0.429
	DT	0.123	0.380	0.409	0.250	0.222	0.235
	GBDT	0.299	0.436	0.591	0.500	0.556	0.526
	Ada	0.047	0.641	0.636	0.545	0.667	0.600
	LR	0.048	0.641	0.591	0.500	0.556	0.526
	NB	0.141	0.675	0.636	0.556	0.556	0.556
	DA	0.282	0.462	0.455	0.385	0.556	0.455
	Mean ± Std	0.121 ± 0.095	0.590 ± 0.144	0.609 ± 0.123	0.524 ± 0.143	0.556 ± 0.182	0.533 ± 0.155

According to Tables 3, 7, the AUC achieved by the CombinedF group across all models is higher than that of the PerfusionF group, except for the NB model. Conversely, the RSD achieved by the CombinedF group across other models is lower than that of the PerfusionF group, except for the RF and Ada models. For the remaining four metrics (Acc, Pre, Recall, and F1), the CombinedF group generally outperformed the PerfusionF group with some exceptions in specific models. The Mean–Variance values in the last column support this, with the CombinedF group achieving Acc, Pre, Recall, and F1 scores of 0.686 ± 0.118, 0.630 ± 0.145, 0.645 ± 0.155, and 0.628 ± 0.123, respectively, outperforming the PerfusionF group’s scores of 0.609 ± 0.123, 0.524 ± 0.143, 0.556 ± 0.182, and 0.533 ± 0.155. This suggests that the prediction performance of perfusion parameter features with the addition of the source features is significantly improved.

Figure 8 illustrates the results of the six metrics for the source features across 10 models. The results highlight that models developed with the 3PWI and MTT feature groups achieved the best predictive performance. The 3PWI feature group attained the highest AUC of 0.863, outperforming all the single-parameter features. This AUC shows an improvement of 16.2, 17.1, 4.2, and 20.9% over the best AUCs for CBV, CBF, MTT, and Tmax, respectively. The AUC values of 3PWI were better than the CBV, CBF, and Tmax groups except on the DT model. The AUC values of 3PWI were superior to the MTT group except on the KNN, DT, and NB models. The other four indices (Acc, Pre, Recall, and F1) had similar patterns. It concluded that the radiomics features of DSC-PWI sequences at the three key time points might be superior to those of individual perfusion parameters in predicting AIS prognosis.

## 5 Discussion

Radiomics technology is a powerful tool for extracting high-throughput quantitative feature information from images, which can extract complex information that is difficult to recognize and quantify by the human eye (37). Using this information, potential prognostic indicators can be identified to construct a prediction model for AIS prognostic outcomes, which can be realized to accurately predict a patient’s prognosis. A prognostic prediction model ultimately helps doctors’ understanding of patients’ prognostic risks and provides an objective basis for assisted decision-making to develop personalized patient treatment plans. In some studies, the prediction of prognostic outcomes has been realized by analyzing medical images (CT, DWI, SWI, PWI, CTP, and others) through radiomics techniques (38–40). Research in Tang et al. (41) has focused on predicting outcomes for AIS by extracting radiomics features based on images post-processed from perfusion sequences. However, there is a lack of studies directly processing the perfusion sequences using radiomics techniques. Another study Guo et al. (17) focused on time-dependent information in the perfusion sequences and analyzed the sequences for all scan times. Still, this approach has led to issues with feature redundancy and excessive computational demands. In this study, we focused on several key time points within DSC-PWI sequences and constructed predictive models using features obtained by radiomics techniques. The final results showed that the radiomics features at three key time points (3PWI) were the most effective for AIS prognostic prediction. In addition, we explored the performance of radiomics features of perfusion parameter maps in predicting AIS outcomes, including single perfusion parameters (CBV, CBF, MTT, and Tmax) and combined perfusion parameters (PerfusionF). The results showed that PerfusionF had an AUC score of 0.889, superior to the single perfusion parameter. In contrast, MTT had the best predictive performance among the four perfusion parameters, with an AUC score of 0.821. Additionally, by combining 3PWI_F and PerfusionF, we developed a CombinedF prediction model that attained an AUC score of 0.915, improving its prediction accuracy. This research confirms that the DSC-PWI sequence contains valuable prognostic-related information and suggests that analyzing only the perfusion parameters obtained by post-processing may lose some essential prognosis-related information.

### 5.1 The DSC-PWI sequences at three key time points provide greater predictive value

DSC-PWI imaging injects a magnetic contrast agent intravenously, altering the blood’s sensitivity to the magnetic field. This causes a change in the intensity of the magnetic resonance signal, which in turn modifies the grayscale of the voxels in the resulting images. In AIS, perfusion is severely affected in areas of insufficient blood flow due to cerebral vascular obstruction, characterized by reduced blood flow and delayed reperfusion. In DSC-PWI sequences, these regions typically display a lower intensity with reduced magnitude and slower response than normal tissue (26). The DSC-PWI sequence provides valuable information about blood flow status and is closely linked to AIS prognosis. However, DSC-PWI images usually contain dozens of time series, each with multiple slices, making feature extraction computationally intensive. Moreover, dozens of DSC-PWI sequences from the same patient contain consistent anatomical information; whether all sequences are necessary for analysis remains an important problem for further investigation. Therefore, multiple critical moments occur when the contrast agent enters and exits these regions; our study explores sequences with one critical time (1PWI), sequences with three critical times (3PWI), sequences with five critical times (5PWI), sequences with seven critical times (7PWI), sequences with nine critical times (9PWI), and all sequences with 50 critical times (50PWI) in assessing their impact on radiomics features for predicting AIS prognosis. The final experiment revealed that the average AUC scores of the six groups across the 10 ML models were ranked as follows: 3PWI > 9PWI > 5PWI > 50PWI > 7PWI > 1PWI, and the best AUC score was obtained by 3PWI on MLP as 0.863, which is a 9.4% improvement over the best AUC score obtained by 50PWI.

The results indicate that the three selected time points—baseline time, peak enhancement time, and perfusion washout time—provide the highest predictive value for AIS prognosis, whereas incorporating additional time points may be counterproductive. In terms of feature extraction and selection, using only three time points significantly reduces computational complexity, by an order of magnitude compared to utilizing all 50 time points. Moreover, all DSC-PWI sequences from the same patient share identical anatomical structures. Repeatedly extracting the same features across multiple time points introduces redundant information. During feature selection, redundant features may be retained along with other correlated features, even if their contribution to prediction is negligible. This not only reduces the efficiency of feature selection algorithms but also degrades the overall performance of the final model. This is because strong correlations may exist among redundant features, potentially leading to collinearity issues. Collinearity can make the coefficient estimates in regression models unstable, resulting in inaccurate predictions (42). Additionally, each sequence contains some level of noise, and incorporating more time-series features may amplify this noise. As a result, the likelihood of retaining noisy features during selection increases, ultimately degrading the performance of the final model. In current literature (43–45), these three time periods of the perfusion curve are highlighted, and several important parameters (Tmax and others) are also derived from the values of these three time periods. The baseline time indicates the moment before the contrast agent arrives and can be used as a benchmark. The peak enhancement time indicates when the contrast agent is actively circulating, which can provide more information about cerebral vascular circulation, and the perfusion washout time indicates that the contrast agent has passed through and is partially absorbed by the tissue, providing valuable information about brain tissue metabolism. Therefore, these three key time points may capture more dynamic changes that are closely related to disease prognosis. The substantial reduction in the initial number of features at these critical time points allows feature selection to focus more effectively on a smaller set of high-quality features. This not only improves the efficiency of model training but also reduces the risk of overfitting. Therefore, analyzing all the sequences is unnecessary, increasing the workload and possibly decreasing prediction performance.

Recent studies have found that perfusion parameters visualize the ischemic condition of brain tissues and reflect the degree of blood–brain barrier (BBB) damage to a certain extent, making them valuable for diagnosis and prognosis in AIS (46). This study explored the prognostic predictive performance of four perfusion parameters (CBV, CBF, MTT, and Tmax) in AIS. The final results were as MTT > CBV > CBF > Tmax, in which the AUC value of MTT was higher than that of the other three parameters across 10 ML models. Its optimal AUC reached 0.821 (DA), with a mean AUC of 0.775, surpassing CBF, CBV, and Tmax by 15.3, 17.7, and 20.3%, respectively. In this study, MTT is considered to play a more significant role in predicting the prognosis of AIS among the four perfusion parameters. However, this may be due to MTT’s higher sensitivity to the ischemic penumbra (ROI). Further research is needed to validate whether MTT is indeed the optimal predictive parameter. Additionally, it is observed that the predictive performance of the models based on the 3PWI generally surpassed the four individual perfusion parameters. For example, on MLP, the optimal AUC of the 3PWI group was still 6.8% higher than that of the MTT group which was the best of the four parameters. Except for the DT model, the 3PWI group outperformed the CBV, CBF, and Tmax groups. These findings suggest that we cannot ignore the rich information of the DSC-PWI sequence itself, and even in AIS prognostic prediction, utilizing the source features of the DSC-PWI sequence may be superior to the post-processed perfusion parameter features.

### 5.2 The feature combination strategy can optimize prognostic prediction performance

Considering that the four perfusion parameters provide different targeted blood flow and tissue information—such as the CBV indicating the ischemic core size, CBF reflecting neuronal activity in ischemic tissue, MTT providing an indirect measure of cerebral perfusion, and Tmax indicating the residual function of the penumbra. This research combined four parametric features to obtain the PerfusionF experimental group. The PerfusionF group achieved an AUC of 0.889 on SVM, significantly better than the four single-parameter features. It indicates that effective integration of relevant information enhances and helps to assess the prognostic results better. Meanwhile, considering the anatomical information in the DSC-PWI sequence, which complements the perfusion parameters, this study continued to combine the perfusion parameter features with the 3PWI features to obtain the CombinedF experimental group. This approach achieved the best AUC of 0.915 using SVM, outperforming all other experimental groups, and is the recommended method in this study. These findings further suggest that the DSC-PWI sequence contains valuable prognosis information related to AIS, emphasizing the need to analyze perfusion parameters and anatomical data. Furthermore, combining multifaceted information can make the information more comprehensive, ultimately improving the accuracy of predicting the prognostic outcome of AIS.

### 5.3 Predictive performance is influenced by the selected model

Machine learning (ML) employs computer algorithms and mathematical models to learn patterns from large amounts of data and make predictions in unknown data. The ML application has penetrated many aspects of disease diagnosis, image analysis, treatment decision-making, and disease prediction (47–50). However, different ML models exhibit varying levels of sensitivity to features. For example, linear models such as Logistic Regression (LR) are more sensitive to linear relationships among features. In contrast, models such as Random Forest (RF) and Decision Tree (DT) are more capable of capturing nonlinear relationships between features. Our research also supports this observation. For example, the DT model achieved AUC by the 3PWI experimental group is only 0.393, lower than all other source feature groups and single-parameter feature groups. Conversely, across other ML models, the 3PWI_F experimental group performed significantly better than other experimental groups, achieving the highest AUC with the MLP model. It indicates that the ML model strongly influences the prediction results, and it is crucial to choose an appropriate ML model.

### 5.4 Limitations and future implications

This study has some limitations that require further optimization work. First, the dataset used was limited and from a single medical center, which includes patients with different treatment strategies but does not account for the latest treatments. This could affect the prognosis of patients. Thus, validating our methodology using a broader and more diverse dataset remains essential before applying it to clinical trials for future work. Second, there was a significant imbalance in the proportion of men and women in the dataset. Although it has been demonstrated previously that gender has a minimal effect on the prognosis of stroke patients after intravenous thrombolysis (51), expanding the dataset could minimize this effect in the future. Finally, exploring the optimal number of key time points, we only considered 1, 3, 5, 7, 9, and 50 time points. It concluded that radiomics features at three key time points yielded the best prognostic predictions for AIS. However, further investigation is needed to determine whether increasing the number of time points beyond three would result in superior outcomes.

Exploring the prognostic value of DSC-PWI sequences at different time points, starting from the perfusion curve, offers Implications for future research. First, this approach provides clinicians and translational researchers with a new perspective to uncover potential pathologies. Second, our results show that DSC-PWI sequences at three key time points provide the best prognostic performance. These time points reflect different stages of the pathological process and allow for more accurate assessment of cerebral blood flow changes. In future studies, beyond these three key time points, additional time points or new perfusion parameters could be explored to evaluate their clinical significance in various pathological conditions. Lastly, in clinical practice, future research could focus on optimizing scanning strategies based on the performance of these key time points. Reducing scans at lower-value time points could save patients’ time and improve diagnostic efficiency. In conclusion, we hope this study will inspire future researchers to continue innovating and develop more efficient tools for clinical use, further advancing personalized medicine and precision treatment.

## 6 Conclusion

This study explored the performance of radiomic features derived from DSC-PWI sequences at different time points and various perfusion parameters in predicting the prognosis of AIS. We found that the prediction performance of the three key time points DSC-PWI (3PWI) outperformed traditional perfusion parameters, highlighting the significant value of the 3PWI features in prognostic prediction. In addition, we observed that the combined four perfusion parameters showed a marked improvement over individual perfusion parameters, suggesting that the integration of effective information allows for a more comprehensive assessment, thereby enhancing predictive performance. Consequently, we combined the 3PWI features with perfusion parameter radiomics features to construct the CombinedF prediction model, which showed even better performance. The AUC reached 0.915, representing a 5.2% improvement over the 3PWI features alone. The proposed method achieves accurate prediction of the prognosis of AIS patients. Furthermore, it could be an objective tool to guide clinical assessment of the prognosis of AIS patients, which has specific application value in helping clinicians develop personalized treatment plans.

## Data Availability

The raw data supporting the conclusions of this article will be made available by the authors without undue reservation.
